# Spatial characteristics of pitch-passing networks under four situational variables in the UEFA Women's euro 2025: a social network analysis

**DOI:** 10.3389/fspor.2026.1839954

**Published:** 2026-06-11

**Authors:** Yingling Luo, Tao Quan

**Affiliations:** College of Physical Education and Health Science, Chongqing Normal University, Chongqing, China

**Keywords:** competition stage, match outcome, match period, pitch-passing networks, score state, social network analysis, spatial characteristics

## Abstract

This study utilized social network analysis to explore the spatial characteristics of pitch-passing networks under four situational variables in the UEFA Women's Euro 2025, including match outcome, competition stage, score state, and match period. The pitch was divided into 48 distinct zones, and six network metrics were calculated. The results indicated that winning teams construct more efficient mediating links within pitch-passing networks, with a stronger reliance on flank-area utilization. From the group to the knockout, passing hubs shift from the defensive third toward the middle third and transitional zones, alongside enhanced local stability and circulation in the attacking third. Competitive pressure further reshapes the contribution of spatial regions to ball progression and network accessibility. In different score states, leading teams tend to maintain structured possession corridors in the defensive third to control risk, whereas trailing teams concentrate activity in central areas, exhibiting higher midfield intensity but reduced stable local passing structure. A significant decline in the physical capability of the pitch-passing networks was observed, with the 1st half characterized by broader distribution and stronger structural integration, and the 2nd half showing reduced connectivity and local intensity. Overall, this study provides actionable and targeted practical guidance and performance analysis. Coaches are encouraged to prioritize flank-area utilization through drills incorporating diagonal runs, wide-area link-up play, and rapid passing along the sideline, while promoting efficient ball circulation in the final third via small-sided and constrained training tasks.

## 1 Introduction

Over the past several decades, performance analysis has become an important component of sports science. Sport performance analysis techniques help coaches, athletes, and sport scientists develop an objective understanding of actual sport performance (1). In football, the continuous development of match event data and player tracking technologies has substantially enhanced researchers' ability to analyze collective team behavior during matches (2, 3). With the accelerating professionalization of women's football and its growing international influence, women's football has received increasing attention in recent years. However, performance analysis still relies heavily on data from men's football, which restricts our understanding of sex-specific playing patterns (4). Therefore, conducting dedicated performance analysis research on women's football has become increasingly necessary. Furthermore, due to substantial physiological, physical, and tactical differences between men's and women's football (5), the passing structures and spatial interaction patterns observed in men's matches may not accurately represent the tactical characteristics of women's football. Previous studies have shown that compared with men's football, women's football exhibits distinct characteristics in match intensity, high-intensity running demands, and patterns of physical confrontation (6). Women's football also differs from men's football in terms of passing choices; research indicates that women's matches typically feature more direct passing, less off-the-ball movement, and less pressing (7). Female players make fewer disposals (passes) to teammates, commit more errors, and contest ball possession more frequently (8). Nevertheless, related research has shown that the technical performance of the athletes in the women's league is different from that in the men's league, although some of these differences appear to be narrowing (3). The differences between the sexes in the style of play in football are due to external physical factors, which lead to logical, strategic adaptations by the female players (5). Thus, against the backdrop of increasing physical demands and a gradual convergence toward higher levels of physical contest similar to those observed in men's football, examining how women's teams establish interactive passing structures and utilize space within offensive and defensive organization to collectively generate tactical advantages during matches represents a topic of considerable research value.

Within team sports, understanding the cooperative and competitive interactions among players has become a central focus of performance analysis (1). In football, passing represents the most frequent form of interaction during match play and reflects the relational structure that underpins collective team organization (9–11). Social network analysis (SNA) has therefore been increasingly adopted as an analysis framework to quantify interaction patterns between players (2). Traditional passing studies conceptualize players as nodes and passes as relational ties, enabling researchers to evaluate information flow, tactical organization, and playing style within teams (12–16). Previous studies categorized applications of SNA in football matches into descriptive, correlational, comparative, and predictive analytical types (17). Comparative articles primarily focus on comparative analyzes based on situational constraints, player positions and team performance (17–20). With regard to the selection of social network analysis metrics, previous studies have predominantly employed degree centrality, closeness centrality, betweenness centrality, and the clustering coefficient. Other metrics used include reciprocity, eigenvector centrality, the associativity coefficient, and zonal interaction patterns; the choice of metrics varies depending on the specific research focus. Football is inherently a spatially structured sport in which player positioning and ball circulation are closely linked to the occupation and control of pitch areas (21, 22). Player-based passing networks capture interpersonal relationships (23), but often overlook the spatial dimension of tactical organization. To address this limitation, recent studies have introduced pitch-passing networks, which divide the playing field into predefined zones and construct networks based on passes exchanged between these areas (24). In this framework, pitch zones are treated as nodes, and the connections between them represent the frequency of passes from one zone to another (*ω*_i_, _j_) (25, 26). This spatial approach allows researchers to identify influential areas of the pitch and to examine how teams utilize space to progress the ball and penetrate defensive structures (27–29). Consequently, spatial passing networks provide a more comprehensive perspective on tactical organization and collective performance during matches (30–32).

While spatial passing networks offer important structural insights, team behavior is further shaped by situational variables such as match stage and score state (15, 33, 34). According to complex systems theory and situational effects theory, football matches are inherently dynamic (34–38). These situational variables can be broadly categorized into time-related and outcome-related conditions. Time-related variables include competition stage (e.g., group stage vs. knockout rounds) and match period (1st half vs. 2nd half), which influence player fatigue, tactical decision-making, and game tempo (39–42). Outcome-related variables include match outcome (43, 44) and score state (45–50). Previous research has indicated that women's football leagues tend to be more competitively unbalanced than men's leagues, potentially reflecting broader structural disparities that extend to international competitions (51). Whether in terms of running performance or match performance, women's football is increasingly displaying certain distinctive characteristics. Some studies have found that women cover more distance than men at lower speeds, especially in the final minutes of the first half (5). Women's matches have, on average, more free kicks, duels, others on the ball, and passes but fewer fouls than men's matches (52). Examining different situational variables contributes to a more comprehensive understanding of teams' behavioral patterns and strategic adjustments under varying match conditions. In particular, under adverse situations, coaches and performance analysis can implement targeted tactical adjustments during both pre-match preparation and in-game management, thereby promoting the stability of team performance (53, 54). However, most existing studies have primarily focused on men's competitions. Research integrating spatial passing networks with multiple situational variables, particularly within elite women's football tournaments, remains relatively limited (37, 55, 56). Therefore, the present study constructs spatial passing networks based on successful passes and analyzes data from the UEFA Women's Euro 2025. Specifically, it investigates how spatial passing structures vary under different situational conditions, including match outcome, competition stage, score state and match period. By doing so, this study aims to examine passing performance in women's football under different situational contexts and to provide new empirical insights into spatial tactical choices in the sport.

## 2 Materials and methods

### 2.1 Dataset description

To address the research objectives, a publicly available and well-documented dataset was utilized. All event data were obtained from Statsbomb open-access data repository, hosted at https://github.com/statsbomb/open-data. In the present study, draw situations were excluded from the analysis. The decisions regarding the treatment of draw situations were made at two distinct levels and are therefore clarified separately. First, at the level of score state, the analysis focused exclusively on leading and trailing situations, while leveling states were excluded. This decision was based on the fact that leading and trailing conditions are more likely to reflect moments of tactical adjustment and strategic adaptation under competitive pressure. In such situations, teams tend to modify their risk-taking behavior and spatial organization, which can be captured through changes in pitch-passing structures. In contrast, although there were plenty of passes even when the score state was level, these passes did not break the deadlock or create any advantage. The states of level generally represent a more balanced and less polarized competitive context, in which tactical behaviors are comparatively stable and less distinctly differentiated. As a result, including draw states may reduce the sensitivity of the analysis to detect contrasting tactical responses. Second, at the level of match outcome, matches that ended in a draw were excluded from the dataset. This decision was guided by both methodological considerations and consistency with prior research. Previous studies in football performance analysis have frequently adopted a binary classification of match outcome (win vs. loss) to maximize contrast between groups and to facilitate the identification of performance differences (35, 51). Following this approach, the present study focused on clearly differentiated competitive outcomes to enhance the interpretability of the results. In addition, drawn matches may reflect a wider range of contextual and tactical conditions, as they can arise from diverse match dynamics (e.g., balanced play, conservative strategies, or late equalizing events). This heterogeneity makes it more difficult to associate draw outcomes with consistent tactical patterns. Therefore, excluding drawn matches helps reduce variability unrelated to the main analytical focus and allows for a clearer examination of differences in pitch-passing structures between winning and losing performances.

Meanwhile, only passes successfully completed by teammates are included in the sample, passes that are intercepted or result in a turnover are excluded. Extra time was excluded from the analysis to ensure comparability across matches and maintain consistency in the definition of situational variables. Extra time only occurs in knockout matches and is characterized by substantially different physiological and tactical conditions compared with regular time, which may introduce situational bias into the analysis. Thus, regular match time was defined as the 90 min of play plus 1st and 2nd half stoppage time. Finally, to ensure the reliability of network metrics derived from passing data, an additional filtering procedure was applied to address potential instability caused by low-pass-count observations. Specifically, score state segments with a duration of less than 10 s were excluded, as such short intervals typically contain very few passes and may lead to unreliable estimations of global network measures (e.g., betweenness centrality and closeness centrality).

Finally, to account for differences in exposure time across score states, strength-based metrics (in-degree, out-degree, and strength) were normalized by the duration of each state. Specifically, these metrics were expressed as pass frequency per minute, ensuring comparability across conditions with unequal observation periods. The duration of each score state was first calculated for each match using Equation 1:

$$T_{m,s}=\frac{\max(t_{m,s})-\min(t_{m,s})}{60}$$ (1)

where $T_{m,s}$ represents the timestamps of all events occurring under score state s in match m, and the result is expressed in minutes. Subsequently, strength-based network metrics were normalized by the corresponding duration, as shown in Equations 2:

$$\mathrm{ID}C_{\mathrm{norm}}=\frac{{\mathrm{DC}}_{m,s}^{\mathrm{in}}}{T_{m,s}},\;\mathrm{OD}C_{\mathrm{norm}}=\frac{{\mathrm{DC}}_{m,s}^{\mathrm{out}}}{T_{m,s}},\;ST_{\mathrm{norm}}=\frac{ST_{m,s\;\;\;\;\;\;\;}}{T_{m,s}}$$ (2)

This normalization expresses passing activity as frequency per minute, ensuring comparability across score states with unequal durations. In contrast, structure-based network metrics, including betweenness centrality, closeness centrality, and clustering coefficient, were computed from the original weighted networks without normalization, as they reflect topological properties of the passing network rather than absolute interaction volume.

The dataset contains detailed information on every pass occurring during matches, including the following key indicators: (i) Timestamp: the time at which the event occurs within the match, recorded to the millisecond. (ii) Period: the match segment to which the timestamp corresponds (1 = first half, 2 = second half). (iii) Location: An array of two integer values representing the x and y pitch coordinates of the event (displayed only for events with valid pitch coordinates). For passing events, the start coordinates denote the passing player's position and the end coordinates denote the receiving player's position. A comprehensive summary of these variables is presented in Table 1.

**Table 1:** Example of the match event data structure.

Match id	Period	Timestamp	Start location	End location
f83bbb5	1	00:00:00.681	[61.0,40.1]	[46.6, 41.5]
95d157e	1	00:00:18.777	[16.9,80.0]	[34.5, 70.9]
c7bda52	1	00:00:35.281	[87.4,0.1]	[108.1, 19.7]
…	…	…	…	…
ec5a01f	2	00:51:09.152	[55.5,24.3]	[65.7, 24.5]
7064daf	2	00:51:16.409	[64.7,25.7]	[65.3, 56.5]
f3a9cce	2	00:51:49.723	[77.5,8.8]	[90.6, 5.8]

The final sample consisted of all 31 matches of the UEFA Women's Euro 2025, yielding a total of 9,547 successful passes. The sample size statistics are shown in Table 2. Furthermore, as the raw data did not include the required situational variables, four situational variables were manually added using Microsoft Excel, including: (i) match outcome (win vs. lose), (ii) competition stage (group vs. knockout), (iii) score state (leading vs. trailing), and (iv) match period (1st half vs. 2nd half). The selection of situational variables in this study was informed by both prior research in football performance analysis and theoretical considerations regarding contextual influences on team behavior (29, 30, 33, 34, 37, 38).

**Table 2:** The sample size statistics of passes under four situational variables.

Situational variables	Category	Number of passes	Total
Match Outcome	Win	5,930	9,547
	Lose	3,617	
Competition Stage	Group	5,891	
	Knockout	3,656	
Score State	Leading	5,573	
	Trailing	3,974	
Match Period	1st half	3,006	
	2nd half	6,541	

After that, to ensure the reliability of the observational data, two independent coders analyzed the passing events from the same matches. Each pass event was treated as the unit of analysis and was coded according to four situational variables: match outcome, competition stage, the score state at the moment of the pass, and match period. In cases of disagreement, a third researcher reviewed the coding and resolved discrepancies through discussion and verification. The coding team consisted of two master's students specializing in football performance analysis and one academic supervisor in the same field, all of whom possessed relevant expertise and research experience in football performance analysis. Inter-rater reliability was assessed using Cohen's kappa coefficient. The results indicated a very high level of agreement between operators (kappa = 0.91), demonstrating strong data reliability and quality control.

### 2.2 Pitch coordinates and zone division

**Figure 1:**
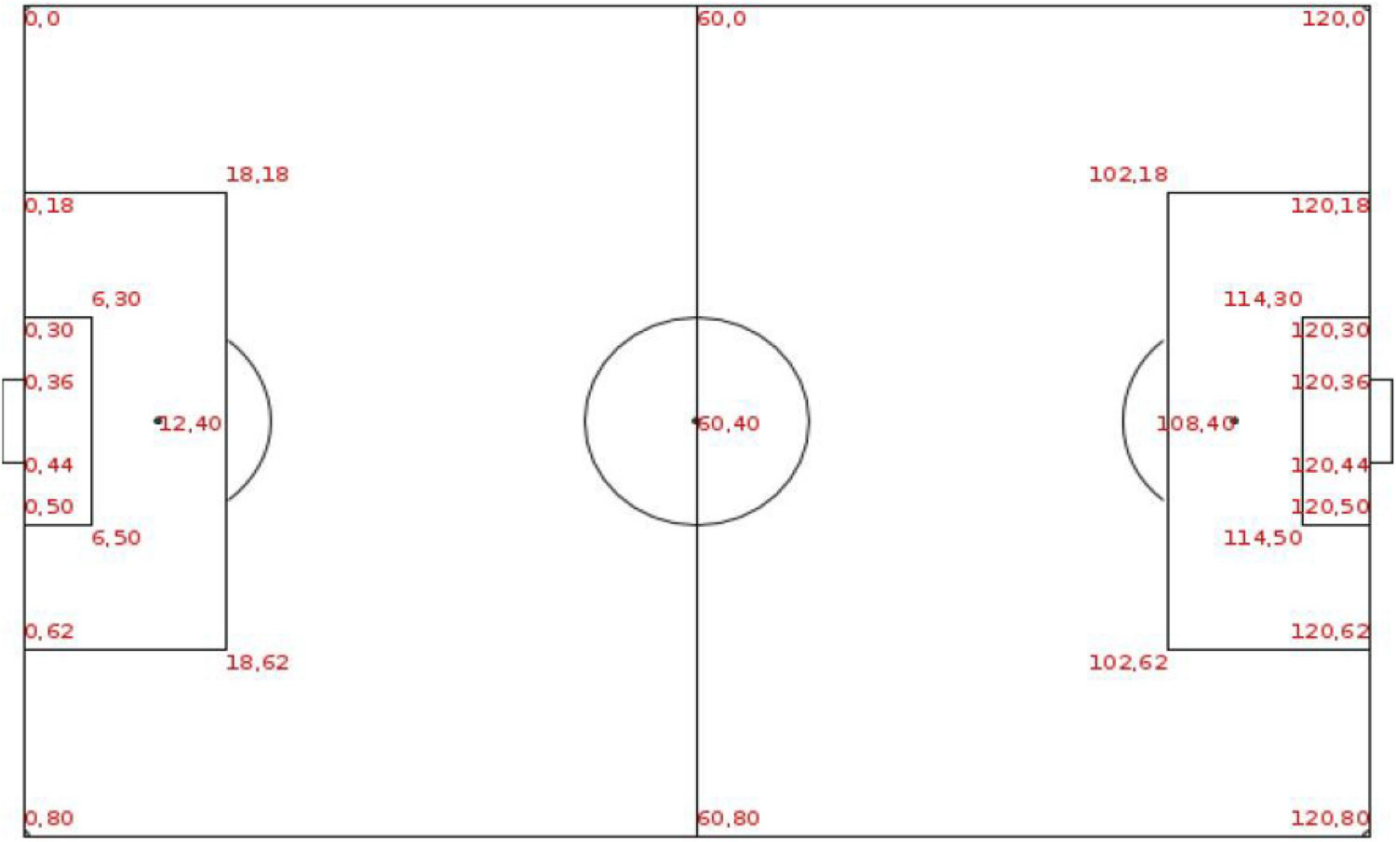
Pitch coordinates specified as (x, y). Reproduced from StatsBomb Open Data documentation (StatsBomb GitHub repository).

According to the coordinate system defined by Statsbomb, the pitch was standardized to a normalized grid, with the *x*-axis ranging from 0 to 120 (attacking direction) and the *y*-axis from 0 to 80 (top to bottom). Figure 1 illustrates the standardized pitch coordinates used for the pitch-passing, which raw data are derived from the official documentation available at the following source: https://github.com/statsbomb.

**Figure 2:**
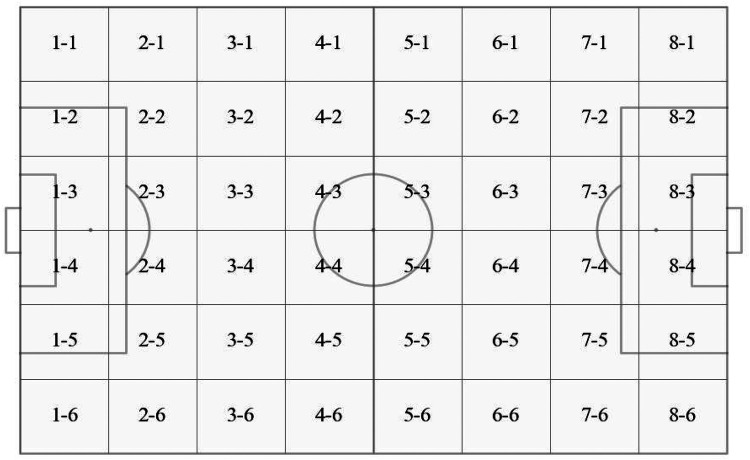
The 48 blocks of pitch zone division (6 × 8).

Previous studies have shown that grid-based passing networks composed of approximately 50 spatial units can capture stable and interpretable passing patterns (25, 29). Based on this evidence, the pitch was divided into 48 equally sized zones. (h = 8, v = 6; 1–1 to 8–6), where h represents the horizontal subdivisions in the x-direction and v represents the vertical subdivisions in the y-direction, with a higher value of “x” meaning proximity to the attacking zone, and a higher value of “y” meaning proximity to the left flank. Each block corresponds to a node, with passes between blocks represented as edges, and the number of passes serving as edge weights. Nodes are designated by the convention column number-row number (e.g., 3–2 denotes the area in column 3, row 2), as illustrated in Figure 2. Compared with symmetric grid structures, the adoption of an asymmetric grid better reflects the inherent rectangular geometry of the football pitch. Specifically, it provides higher spatial resolution along the longitudinal direction, which is critical for capturing tactical behaviors such as ball progression, penetration, and transitions between defensive and offensive phases. Meanwhile, a relatively coarser partition in the lateral direction helps avoid excessive spatial fragmentation, thereby enhancing the robustness and interpretability of network-based metrics. When a pass occurs from block i to block j, a link is created from node i to node j, with a weight assigned to quantify the total number of completed passes in that direction. This approach converts complex contiguous spaces into discrete network nodes, thus facilitating subsequent computations.

### 2.3 Social network analysis

After partitioning the football pitch, all passes were mapped to their corresponding zones, yielding the starting zone and ending zone for each pass. The number of passes within each zone and between zones was converted into an adjacency matrix to construct the passing networks. A weighted directed adjacency matrix was constructed using passing events as edges. Values represent the count of successful passes between blocks. Diagonal elements indicate internal passes within a block. For example, read the team's pass data sequentially: if a pass occurs from starting zone i and destination zone j, the value at position (i, j) in the matrix was incremented by 1. The resulting matrix element adj_(ij) represents the number of passes from zone i to zone j. This yielded a weighted directed adjacency matrix and an adjacency matrix was created for each team (as shown in Table 3). All network metrics were calculated on these weighted directed networks, preserving both the direction and frequency of passes between zones. Consequently, directional metrics such as IDC and ODC were computed directly from the directed adjacency matrix. Edge weights were retained to reflect the intensity of passing interactions between zones, rather than being binarized.

**Table 3:** Example data of network adjacency matrix for pitch-passing networks.

Block	1–1	1–2	1–3	…	8–4	8–5	8–6
1–1	1	0	0	…	0	0	0
1–2	2	1	4	…	0	0	0
…	…	…	…	…	…	…	…
8–5	0	0	0	…	0	0	0
8–6	0	0	0	…	1	0	0

Subsequently, social network metrics were calculated separately under different situational variables, allowing for a focused assessment of how these factors shape the spatial structure of passing networks. The analysis framework employed in this study was conceptually informed by the work of Huang et al. (29), who proposed a generalized network-based approach for evaluating spatial passing performance in football. While the present study follows the overall analytical logic of this framework, several substantive and context-driven modifications were introduced to ensure its suitability for the current research context. First, the data source differs entirely, with the present study utilizing Statsbomb data from elite women's international competition. This difference may influence both event definitions and network structures. Second, the pitch grid division scheme was adjusted to better capture spatial characteristics of play, particularly in terms of longitudinal progression and lateral distribution. Third, SNA metrics were expanded to six indicators to capture distinct functional aspects of passing behavior, rather than relying on a more limited set of measures. Fourth, the set of situational variables was revised. Taken together, these modifications represent a targeted adaptation of the original framework rather than a direct replication. The overall framework of this study is as follows: (i) Pitch coordinates and zone division; (ii) Convert the pitch-passing networks into an adjacency matrix; (iii) Construct the pitch-passing networks; (iv) Differences under situational variables.

### 2.4 Network metrics

In this section, the network metrics used in social network analysis are defined. In the pitch-passing networks, a total of 48 nodes are defined, corresponding to the 48 spatial zones into which the pitch is divided, with each zone representing a single node. Considering the potential interdependence among different network metrics, the analysis emphasizes consistent patterns observed across multiple indicators rather than relying exclusively on the statistical significance of individual measures.

#### 2.4.1 In-degree centrality

The in-degree centrality (IDC) quantifies the frequency and importance of passes received by a given zone. A higher IDC indicates greater involvement of a zone in receiving passes within the team's weighted passing network (17), as defined in Equation 3:

$$S_{in}(i)=\sum_{\;j=1}^{N}w_{\;ji}$$ (3)

Where $W_{\;ji}$ represents the number of passes from zone j to zone i (the edge weight).

#### 2.4.2 Out-degree centrality

The out-degree centrality (ODC) quantifies a zone's capacity to distribute the ball to other areas and its organizational role within the team's network. A higher ODC indicates greater involvement of a zone in distributing passes to other areas (17), as defined in Equation 4:

$$S_{\mathrm{out}}(i)=\sum_{j\;=\;1}^{N}w_{\mathrm{ij}}$$ (4)

#### 2.4.3 Strength

Strength (ST) represents the total weighted involvement of a zone in the passing network, integrating both incoming and outgoing interactions. These metrics were retained together due to their distinct interpretative roles. Specifically, ODC reflects a player's contribution to ball distribution, while IDC captures ball reception behavior. In contrast, ST provides an aggregated measure of overall involvement in passing interactions. This distinction enables a more nuanced understanding of directional vs. total participation within the network. Zones with higher ST act as active centers within the team's offensive structure, reflecting the team's spatial control and positional strategy, as defined in Equation 5:

$$\begin{array}{c} \;S(i)=\sum_{j\;=\;1}^{N}\left(w_{\mathrm{ij}}+w_{\mathrm{ji}}\right) \end{array}$$ (5)

#### 2.4.4 Betweenness centrality

Betweenness Centrality (BC) measures the extent to which a zone lies on the shortest paths between other zones. Zones with higher BC act as structural bridges facilitating ball circulation and information flow within the team's passing network.

Importantly, since edge weights represent the number of passes (i.e., larger values indicate stronger connections rather than longer distances), all shortest–path–based computations were performed after transforming weights into distances using an inverse transformation, as shown in Equation 6:

$$d_{ij}=\frac{1}{w_{ij}}$$ (6)

Where $w_{\mathrm{ij}}$ is the number of passes from zone i to zone j. This transformation ensures that frequently used passing connections correspond to shorter distances in the shortest-path computation. A higher BC indicates that a zone plays a critical intermediary role in offensive transition pathways (17), as defined in Equation 7:

$$C_{B}(i)=\sum_{s\neq i\neq t}\frac{\sigma_{st}(i)}{\sigma_{st}}$$ (7)

#### 2.4.5 Closeness centrality

The closeness centrality (CC) measures the reciprocal of the average distance from node i to all other nodes. CC measures the average distance from one zone to other zones. Zones with higher centrality can rapidly engage with other zones, enabling swift participation in attack or defense during the match (17):

To ensure correct interpretation of shortest-path distances, edge weights were transformed. CC was then computed as the reciprocal of the average shortest-path distance, as defined in Equation 8:

$$CC_{i}=\frac{1}{\sum_{\;j\neq i}d(i,j)}$$ (8)

Where d_ij_ denotes the shortest path length between node i and node j.

#### 2.4.6 Clustering coefficient

An important measure of network topology, called the clustering coefficient (CCL), assesses the triangular pattern and the connectivity in a vertex's neighborhood: a vertex has a high clustering coefficient if its neighbors tend to be directly connected to each other (57). Clustering coefficient was introduced more recently as a structural feature characterizing small-world networks (58).

The CCL measures the extent to which a zone forms stable, recurrent triangular passing structures with its neighbors. A higher CCL indicates that the zone participates in consistent passing combinations, exhibits strong ball possession, and actively creates local numerical advantages. This metric is important for evaluating passing playing style (59), as defined in Equation 9.

$$C(i)=\frac{\sum_{\;j,k}{\left(w_{ij}w_{\;jk}w_{ki}\right)}^{1/3}}{k_{i}(k_{i}-1)}$$ (9)

Where $w_{\mathrm{ij}}$ denote the weight of passes between zones i and j, and k_i_ represent the number of adjacent zones connected to zone i.

### 2.5 Statistical analysis

All statistical analysis were conducted to examine differences of pitch-passing under four situational variables. A node-level social network metrics included IDC, ODC, ST, BC, CC, and CCL. A comparative analysis was performed independently for each situational variable. In the present study, the statistical unit of analysis was the match-level observation for each spatial zone under a given situational context, rather than nodes within a single network instance. Therefore, comparisons were performed on distributions of zone-specific metrics aggregated across matches. Nevertheless, spatial zones belong to the same passing network and cannot be considered completely independent in a strict statistical sense. Consequently, the results should be interpreted as identifying spatial tendencies in network organization rather than strictly independent experimental effects.

To determine the appropriate statistical test, the Shapiro–Wilk test was first applied to assess the normality of the combined samples for each pairwise comparison. When the data met the normality assumption (*p* > 0.05), an independent-samples t-test was used to evaluate group differences. For these parametric tests, Cohen's d (60–62) was first calculated and then converted into a correlation-based effect size (r) to ensure comparability across all analyzes. Specifically, Cohen's d values were converted into correlation coefficients using the standard transformation formula (Equation 10):

$$r=\frac{d}{\sqrt{d^{2}+4}}$$ (10)

Conventional thresholds for interpretation were adopted (small = 0.1, medium = 0.3, large = 0.5) (63).

When the assumption of normality was violated, the Mann–Whitney *U*-test was employed. For these non-parametric comparisons, *p*-values were converted into Z-scores, and effect sizes were expressed as correlation coefficients using Equation 11:

$$r=\frac{Z}{\sqrt{N}}$$ (11)

Where N represents the total sample size of the two groups. This unified effect size framework ensures consistency and direct comparability of effect sizes across all zones and situational conditions, regardless of whether parametric or non-parametric tests were applied. These procedures ensured a robust and coherent evaluation of differences across all match situations.

These procedures ensured consistent and rigorous evaluation of differences across all match situations. To address the issue of multiple comparisons across regions and metrics, the Benjamini–Hochberg procedure was applied to control the false discovery rate within each metric × situational variable combination, where the 48 zone-level comparisons constituted a single family of tests. Statistical significance was set at *p* < 0.05. All analyses were conducted in Python using the pandas, NumPy, and SciPy libraries. Network construction and metric computations were performed using NetworkX (version 3.6).

For each network metric, only zones reaching statistical significance (*p* ≤ 0.05) were visualized with color shading, while non-significant zones remained unshaded to emphasize meaningful spatial characteristics. All pitch visualizations were produced using Python 3.13 with matplotlib, mplsoccer, NumPy, and pandas' libraries.

## 3 Results

To make the results more intuitive, we visualized this section of the findings. The overlaid boxplots allow direct visual comparison of metric variations across the same field zones. These figures provide clear and concise representation of differences in network metrics from a macro perspective. The *x*-axis represents the 48 zones of the football pitch (from 1 to 1 to 8–6), and the *y*-axis represents the values of each respective metric. The lower and upper edges of the box indicate the 25th percentile (Q1) and 75th percentile (Q3), respectively; the line inside the box represents the median; whiskers extend to data points within 1.5 times the interquartile range (IQR) from the box; outliers are indicated by small plus symbols “+”. In addition, a line graph has been included to highlight the overall trend. This study presents only a selection of descriptive visualization results (shown in Figure 4). The full set of results can be found in the Supplementary Materials, other overlaid boxplots will be omitted.

### 3.1 Spatial characteristics under match outcome

**Figure 3:**
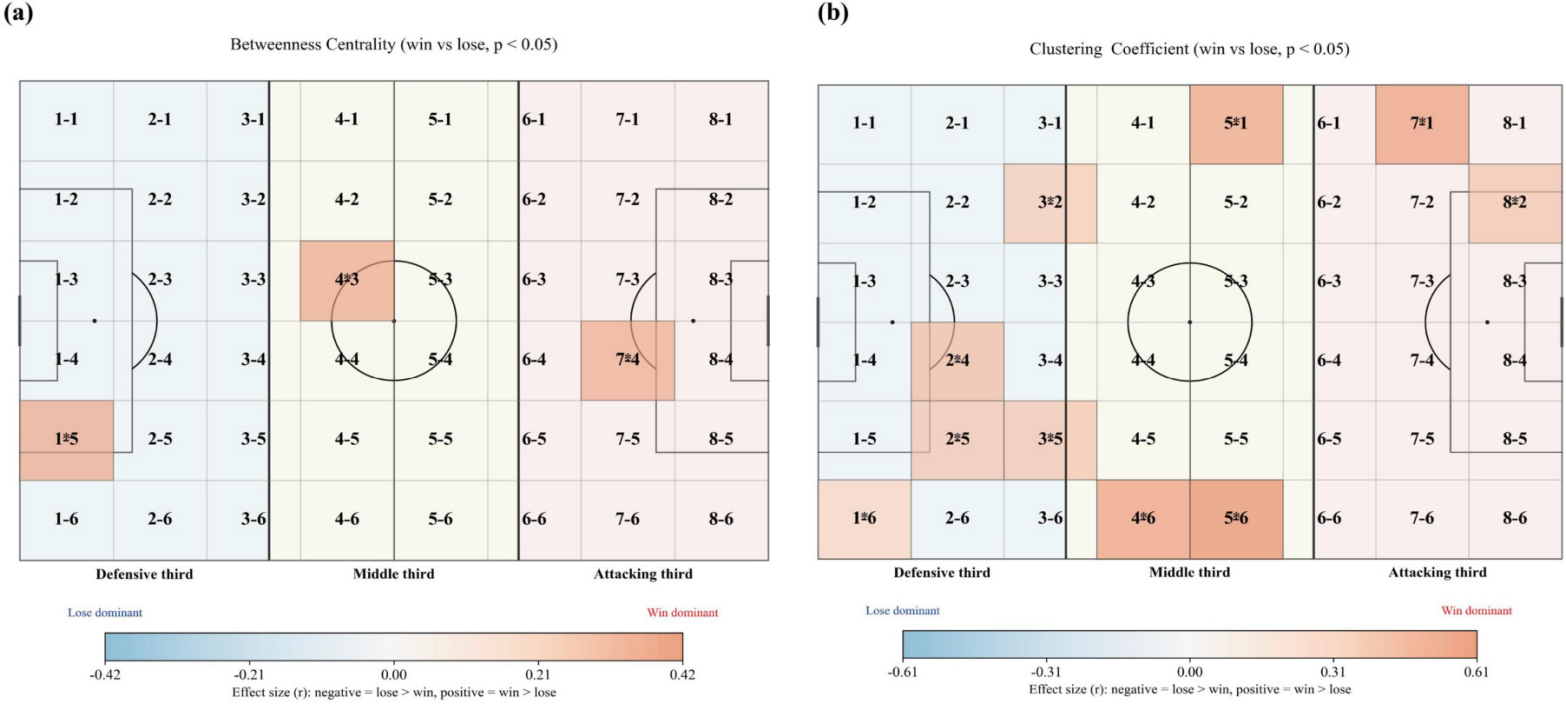
Spatial characteristics of pitch-passing networks under match outcome. (a) Betweenness centrality; (b) clustering coefficient.

Figure 3a indicated that the winning teams exhibited significantly higher BC in several specific pitch zones. These zones were distributed across different thirds of the pitch, including one zone in the defensive third (block 1–5, *p* = 0.022, *r* = 0.419), one zone in the middle third (block 4–3, *p* = 0.039, *r* = 0.420), and one zone in the attacking third (block 7–4, *p* = 0.040, *r* = 0.408).

**Figure 4:**
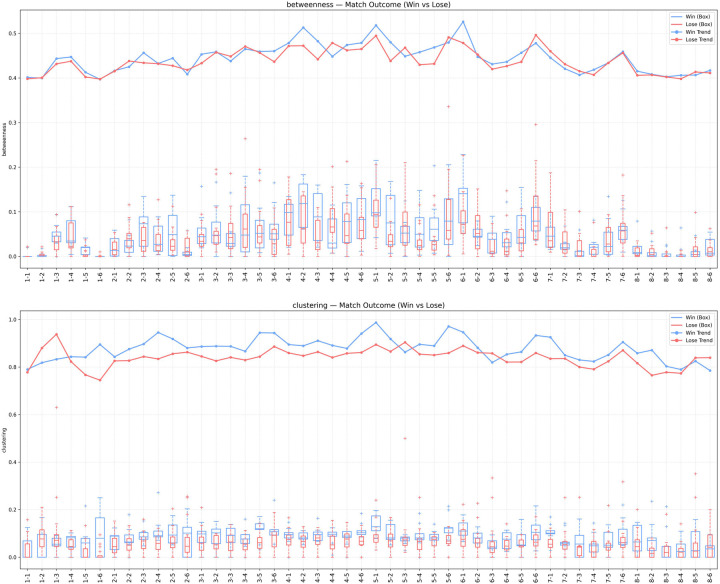
Overlaid boxplots across 48 pitch zones under match outcome.

Figure 3b indicated that the winning teams demonstrated significantly higher CC in multiple spatial zones, with a clear concentration in wide and advanced areas of the pitch. In the defensive third, higher CC values were observed primarily on the right flank, including block 1–6 (*p* = 0.044, *r* = 0.351), blocks 2-(4, 5) (*p* = 0.013–0.029, *r* = 0.442–0.498), and block 3–5 (*p* = 0.025, *r* = 0.453). In the middle third, significant differences were mainly located in wide areas, including blocks (4, 5)-6 (*p* = 0.002–0.007, *r* = 0.543–0.611), as well as block 5–1 (*p* = 0.009, *r* = 0.520). In the attacking third, higher CC values for winning teams were identified in left-flanked zones, including block 7–1 (*p* = 0.007, *r* = 0.543) and block 8–2 (*p* = 0.030, *r* = 0.430). No statistically significant differences were observed for the other network metrics.

### 3.2 Spatial characteristics under competition stage

**Figure 5:**
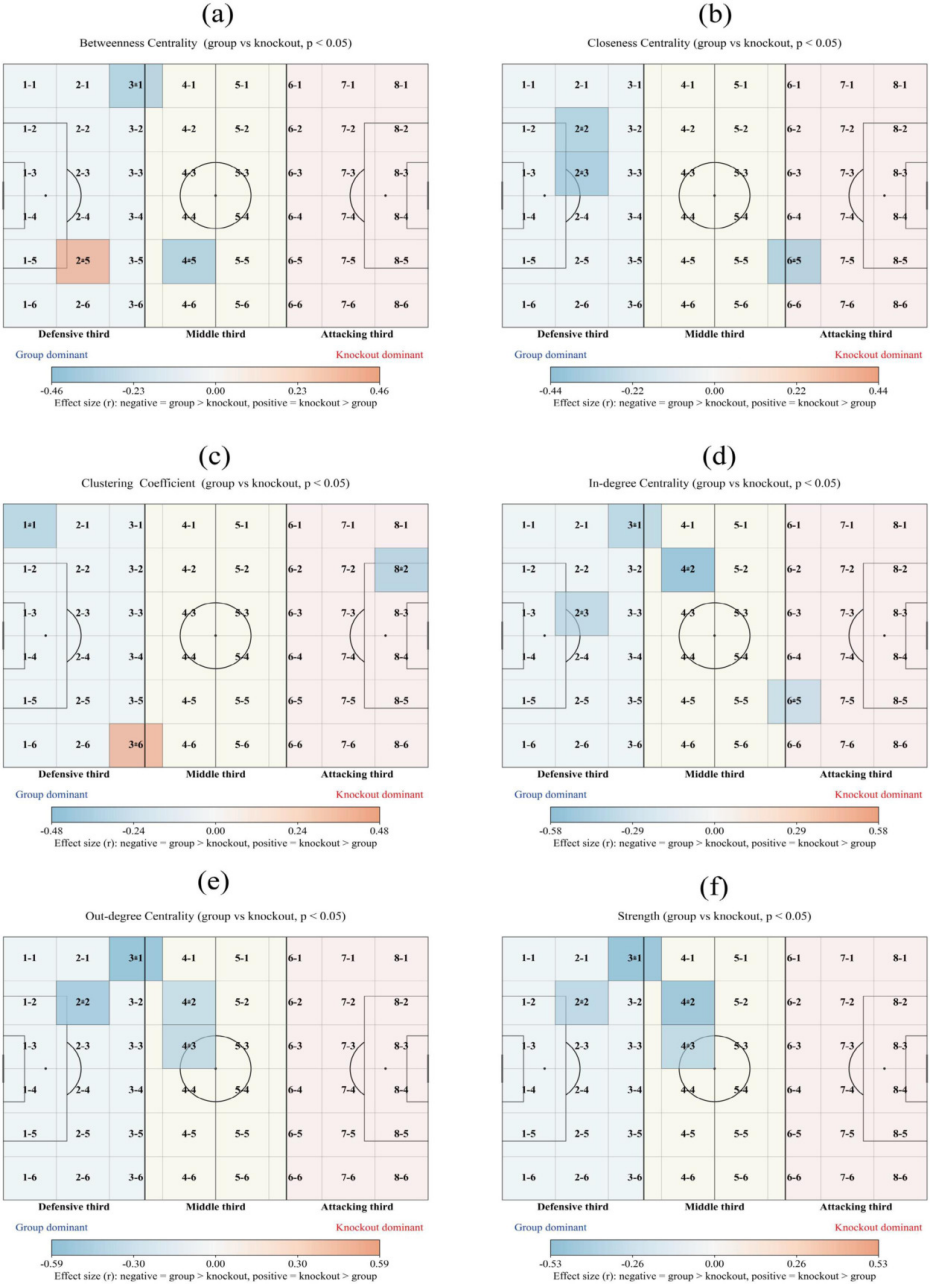
Spatial characteristics of pitch-passing networks under competition stage. (**a**) Betweenness centrality; (**b**) closeness centrality; (**c**) clustering coefficient; (**d**) in-degree centrality; (**e**) out-degree centrality; (**f**) strength.

Figure 5a showed that BC differed between the group and knockout stages in a limited number of zones. Specifically, higher BC values in the group stage were observed in the defensive third (block 2–5, *p* = 0.024, *r* = 0.456). In contrast, during the knockout stage, higher BC values were identified in two zones, including the defensive third (block 3–1, *p* = 0.036, *r* = −0.424) and the middle third (block 4–5, *p* = 0.028, *r* = −0.446). Figure 5b indicated that CC was significantly higher in the knockout stage across several zones. These were mainly located in the defensive and middle thirds, including blocks 2-(2, 3) (*p* = 0.031–0.047, *r* = −0.435–0.403), as well as block 6–5 (*p* = 0.036, *r* = −0.424). Figure 5c demonstrated that IDC was significantly higher in the knockout stage, particularly in the left flank of the defensive third. Significant zones included block 2–3 (*p* = 0.027, *r* = −0.446), block 3–1 (*p* = 0.016, *r* = −0.484), and block 4–2 (*p* = 0.004, *r* = −0.582). Additionally, one zone in the attacking third of the right flank (block 6–5, *p* = 0.049, *r* = −0.397) also showed higher IDC values in the knockout stage. Figures 5d–f further revealed that IDC, ODC and ST were significantly higher in the knockout stage, particularly in the defensive third of the left flank. For example, significant differences in ODC were observed in block 2–2 (*p* = 0.011, *r* = −0.511), block 3–1 (*p* = 0.003, *r* = −0.592), and blocks 4-(2, 3) (*p* = 0.023–0.031, *r* = −0.456–0.435).

### 3.3 Spatial characteristics under score state

**Figure 6:**
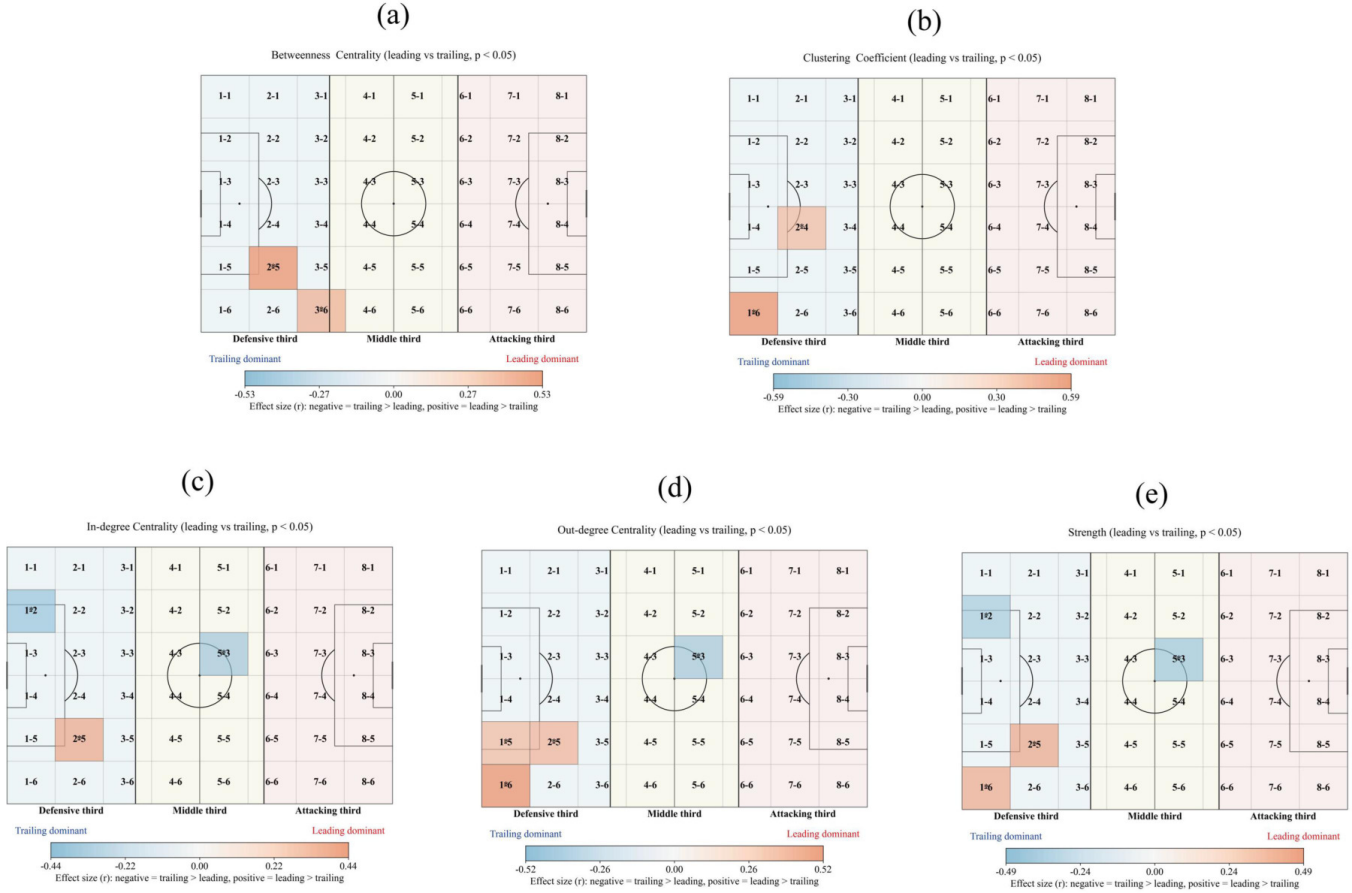
Spatial characteristics of pitch-passing networks under score state. (**a**) Betweenness centrality; (**b**) clustering coefficient; (**c**) in-degree centrality; (**d**) out-degree centrality; (**e**) strength.

Figure 6a shown that teams exhibited higher BC when in a score state of leading. This effect was observed in two zones, including block 2–5 (*p* = 0.004, *r* = 0.534) and block 3–6 (*p* = 0.016, *r* = 0.489). As shown in Figure 6b, when teams were leading, higher CC values were primarily observed in specific zones within the defensive third. This included block 1–6 (*p* = 0.001, *r* = 0.592) and block 2–4 (*p* = 0.020, *r* = 0.474). Results in Figure 6c indicated contrasting patterns between leading and trailing score states. When leading, teams showed higher IDC in block 2–5 (*p* = 0.024, *r* = 0.444). In contrast, when trailing, higher IDC values were observed in block 1–2 (*p* = 0.033, *r* = −0.430) and block 5–3 (*p* = 0.038, *r* = −0.415). Figure 6d demonstrated that, when leading, teams exhibited higher ODC predominantly in the right flank of the defensive third. Significant zones included blocks 1-(5, 6) (*p* = 0.008–0.040, *r* = 0.415–0.519) and block 2–5 (*p* = 0.019, *r* = 0.460). Conversely, when trailing, higher ODC values were identified in a central middle third (block 5–3, *p* = 0.015, *r* = −0.474). Figure 6e showed a similar pattern for ST. When leading, higher values were observed in the defensive third, including block 1–6 (*p* = 0.019, *r* = 0.474) and block 2–5 (*p* = 0.011, *r* = 0.489). In contrast, when trailing, higher ST values were found in block 1–2 (*p* = 0.041, *r* = −0.415) and block 5–3 (*p* = 0.019, *r* = −0.459). Overall, IDC, ODC, and ST consistently indicated that when teams were leading, the defensive third of the right flank (block 2–5) was the most intensively utilized area. In contrast, when teams were trailing, activity was more concentrated around the central midfield zone, especially block 5–3.

### 3.4 Spatial characteristics under match period

**Figure 7:**
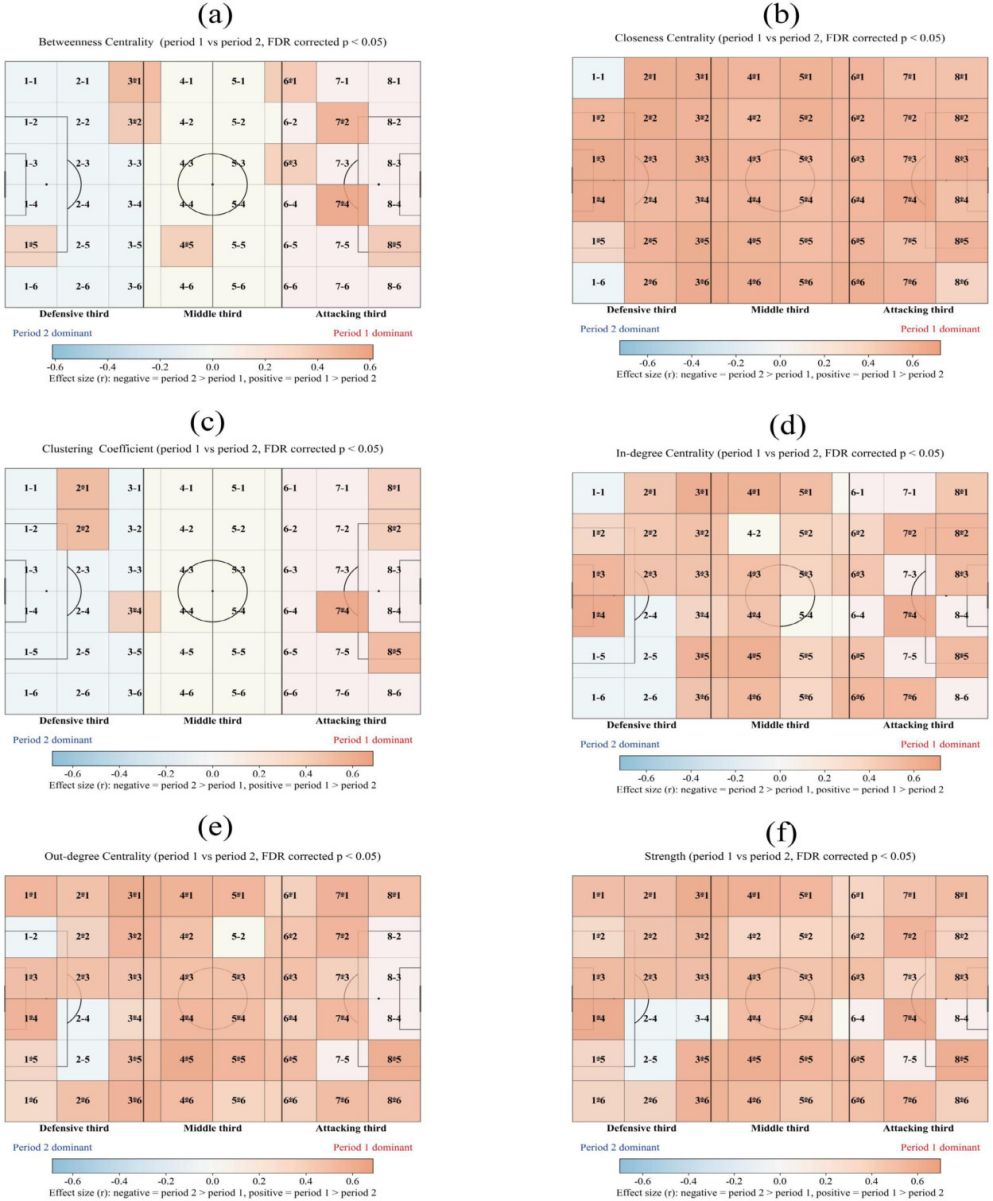
Spatial characteristics of pitch-passing networks under match period. (**a**) betweenness centrality; (**b**) closeness centrality; (**c**) clustering coefficient; (**d**) in-degree centrality; (**e**) out-degree centrality; (**f**) strength.

Figure 7a showed that BC values were generally higher in the 1st half compared to the 2nd half, although the spatial distribution of significant zones was relatively dispersed. Specifically, higher BC values in the 1st half were observed in the defensive third (block 1–5, *p* = 0.039, *r* = 0.420), in the middle third of the left flank [blocks 3-(1, 2), *p* = 0.023–0.024, *r* = 0.490–0.500], and across multiple zones in the attacking third. These included blocks 6-(1, 3) (*p* = 0.027–0.049, r = 0.439–0.483), blocks 7-(2, 4) (*p* = 0.008–0.016, *r* = 0.547–0.610), and block 8–5 (*p* = 0.030, *r* = 0.477). As shown in Figure 7b, CC values were consistently higher in the 1st half across nearly the entire pitch. Significant differences were widespread (*p* = 0.001–0.030, *r* = 0.484–0.710), indicating a global increase in network efficiency during the 1st half. Figure 7c indicates that the CCL was also higher in the 1st half in several key zones. In the defensive third, this included blocks 2-(1–2) (*p* = 0.013, *r* = 0.560–0.570) and block 3–4 (*p* = 0.027, *r* = 0.496). In the attacking third, higher CCL values were observed in blocks 8–1, 8–2, and 8–5 (*p* = 0.010–0.030, *r* = 0.470–0.570), as well as block 7–4 (*p* = 0.005, *r* = 0.687), which demonstrated a particularly large effect size. Figures 7d–f further demonstrate that IDC, ODC, and ST values were generally higher in the 1st half across most areas of the pitch. Notably, strong effects were observed in several key zones, including block 1–4 (*p* = 0.004–0.008, *r* = 0.630–0.719), block 3–1 (*p* = 0.005, *r* = 0.670–0.680), block 4–5 (*p* = 0.005, *r* = 0.680), blocks 7-(2, 4) (*p* = 0.005–0.010, *r* = 0.570–0.680), and block 8–5 (*p* = 0.006–0.010, *r* = 0.590–0.660). Overall, the results consistently indicate that network-related metrics were higher in the 1st half across large portions of the pitch.

## 4 Discussion

By analyzing these variables and calculating network metrics, this study aimed to reveal how situational variables influence and shape the structural patterns and spatial tactical patterns within the game. It also sought to provide valuable insights into team performance and strategic decision-making processes under varying game conditions, as well as to enhance understanding of how external factors shape on-field coordination and tactical execution.

### 4.1 Mediating links and flank-area utilization of passing

When comparing passing networks between winning and losing teams, the results indicate that match outcome is primarily associated with differences in BC and CC. This suggests that performance differences are not primarily driven by the quantity or density of passing interactions, but rather by the structural organization and functional roles of specific pitch zones within the passing network.

BC reflects a zone's role as an intermediary connecting network parts. High BC zones act as key transitional corridors for reorganizing and redistributing ball possession. Winning teams' higher BC across defensive, midfield, and attacking thirds indicates they establish multi-layered transitional pathways instead of relying on a single progression route. This aligns with prior research linking reduced reliance on single passing outlets and more well-connected intra-team passing to enhanced performance (64). The distributed BC pattern implies greater tactical flexibility. By embedding intermediaries across multiple pitch regions, teams maintain possession continuity even when specific passing options are constrained by opponent pressure. Practically, this reduces network fragmentation risk and facilitates smoother progression from build-up to attack. In the context of women's football, where defensive compactness and collective pressing structures are increasingly prominent, the ability to maintain multiple alternative passing routes may be particularly important for overcoming spatial constraints.

CC reveals a distinct spatial advantage for the winning team. The spatial distribution of higher CC values is not uniform but instead concentrated across both flanks. This asymmetrical yet systematic pattern suggests that successful teams do not rely on a single central corridor, but instead construct multiple high-accessibility pathways across lateral channels. This reduces dependence on congested central areas and enables shorter topological distances between key zones during ball progression. Previous studies have suggested that, due to limitations in spatial utilization efficiency, lower-performing teams are more likely to be exposed to higher pressing intensity. Players operating in deeper defensive areas typically experience greater positional security and reduced pressure, which encourages more conservative, possession-oriented passing strategies (28, 65, 66). In contrast, the present findings show that winning teams also exhibit higher local structural stability and tighter combinational connectivity in defensive zones. This suggests that increased clustering in deep areas should not be interpreted solely as a marker of passive or constrained play. Instead, for successful teams, such localized connectivity may reflect a controlled build-up structure, in which stable short-passing configurations are deliberately established to secure possession and organize subsequent progression. In this sense, the defensive third functions not only as a pressure-relief zone but also as an initial platform for structured ball circulation and tactical development. There are obvious differences with the strong central dominance of men (15, 66).

### 4.2 Spatial reconstruction of passing under competitive pressure

The transition from the group stage to the knockout stage is characterized by a selective redistribution of key network functions across specific pitch zones. This indicates that competitive pressure primarily reshapes the functional contribution of distinct spatial regions to ball progression and network accessibility, rather than fundamentally altering the global architecture of the passing system.

In the group stage, higher BC in block 2–5 showed deep defensive areas acting as central transitional hubs for early-phase progression. In contrast, the knockout stage redistributes intermediary functions to defensive and central zones (blocks 3–1 and 4–5), indicating ball progression relies less on a single defensive outlet and more on multiple transition points across deeper and central regions. This suggests a more cautious, spatially balanced build-up strategy with reduced reliance on isolated passing hubs under increased pressure. Higher knockout-stage values concentrate in defensive and middle thirds, reflecting efficient integration of these regions into shorter passing pathways linking defensive build-up and advanced zones, not increased possession frequency. In knockout matches, prioritizing ball security and risk minimization, this may indicate a structured circulation pattern with deeper and central areas as efficient connectors in a compressed, strategic network. Spatial distribution of IDC, ODC, and ST further supports this, highlighting concentrated functional activity in the left defensive third during knockouts. Simultaneous increases in IDC and ODC here suggest dual roles as key receiving and initiating regions in passing sequences, reflecting localized reinforcement of possession stability through structurally important defensive areas under heightened pressure.

In the knockout stage, teams often rely on a smaller defensive third subset for ball circulation and progression, indicating reduced spatial variability and a preference for secure, rehearsed passing structures. This aligns with the strategic demands of the knockout stage, where risk minimization and structural coherence typically take precedence over expansive exploration. Teams construct tighter, more organized passing networks in deeper areas, which serve as both stabilizing hubs and transitional gateways. This spatial reorganization reflects a shift toward structurally efficient, risk-managed ball progression under heightened pressure, where maintaining control in constrained areas becomes essential for advancing into higher-value attacking zones.

### 4.3 Distinct tactical selections under different score state

Leading score state favor the establishment of structurally stable circulation patterns in deeper defensive areas, supporting possession control and risk management. In contrast, trailing conditions shift functional activity toward central hubs, reflecting a strategic need to increase tempo and facilitate more direct access to attacking zones. These findings highlight that score state influences not only the intensity of play but also the spatial logic of team connectivity, shaping how passing networks adapt to the competitive demands of elite women's football.

Under the leading score state, women's teams that maintain high passing density and network connectivity in the defensive third may indicate a more conservative possession strategy. Specifically, these teams appear to construct stable passing structures within low-risk areas to minimize turnovers and manage game tempo. In women's football, where overall duel intensity and explosive speed are generally lower than in men's football, the sustainability of high pressing is often constrained. Maintaining structurally consistent connections between the defensive and middle thirds may therefore represent a risk-management strategy, allowing teams to control tempo while preserving possession security. Importantly, this should not be interpreted as passive play, but rather as a controlled form of progression that minimizes exposure to turnovers in high-risk central zones. Defensive structures remain well integrated within the overall passing network, rather than being isolated as purely protective units.

Under the trailing score state, IDC, ODC, and ST collectively indicated a clear shift of activity toward the middle third (block 5–3). This spatial pattern reflects the attacking intent shifts towards the central passing hub. From a tactical perspective, trailing score state typically require accelerated ball progression and increased occupation of central areas to facilitate quicker access to dangerous zones of the opponent. Previous research in men's football has shown that significantly higher passing frequencies in the middle and attacking thirds under the leading score state (26). However, this is not reflected in the BC and CCL, which may be related to the quality and ability of long passes in women's football. In contrast, the observed shift of passing density toward the midfield likely reflects a more proactive attacking orientation. As the central link between defense and attack, an increase in passing density within midfield zones suggests that teams attempt to accelerate offensive progression by enhancing central penetration and vertical connectivity, rather than relying on relatively low-efficiency wide crosses. This is particularly relevant in women's football, where reliance on wide crossing alone may produce lower direct scoring efficiency compared with the men's game. Consequently, central combination plays and ground-based passing structures may represent more effective attacking solutions.

### 4.4 Physical capability of pitch-passing between the 1st and 2nd halves

During the 1st and 2nd halves, the pitch-passing networks demonstrated significant temporal decay characteristics. In the 1st half, passes were not confined to isolated spatial regions but were distributed across multiple yield areas, in contrast, the overall connectivity and local intensity of the passing network declined in the 2nd half. The 1st half was characterized by higher structural integration, more coherent passing clusters, and broader spatial utilization. As the match progressed, the physical and technical-tactical states of the players gradually deteriorated, leading to a marked reduction in the connectivity of the passing network in the 2nd half.

High BC zones in the 1st half suggest that teams depend on multiple transitional corridors to facilitate ball progression, thereby supporting more diverse and flexible build-up structures. In the context of women's football, this pattern may be associated with greater collective physical availability at the beginning of the match, enabling higher levels of positional mobility and more frequent utilization of both flank and central channels during progression phases. One of the most notable findings is the enhanced network accessibility and more efficient inter-zonal connectivity across nearly the entire pitch of the 1st half. The widespread of this effect suggests that teams are able to circulate the ball more effectively across defensive, middle, and attacking thirds. This pattern is particularly relevant in women's football, where the early phases of matches are often characterized by higher collective intensity and more synchronized pressing and counter-pressing behaviors, leading to faster ball circulation and tighter spatial coordination within the team structure. The stronger local clustering of passing interactions in the 1st half, particularly in both defensive and attacking thirds, suggests that teams are more capable of sustaining coordinated combinational play in the high-value attacking third during the early stages of the match. Importantly, the large effect sizes observed in these zones imply that they may function as key structural hubs for attacking third combinations, where compact support structures facilitate penetration and chance creation. Similarly, the consistent 1st half dominance in IDC, ODC, and ST indicates higher levels of involvement in ball reception, distribution, and overall spatial utilization intensity during the early phases of play. The concentration of strong effects in central and wide attacking corridors suggests that early match attacking structures are more expansive and territorially aggressive.

In the 2nd half, the systematic reduction of these network metrics likely reflects the combined influence of physiological fatigue, tactical adaptation, and situational constraints. Declines in high-intensity running capacity may impair players' ability to maintain compact positional structures and synchronized passing movements, thereby reducing overall network connectivity. Previous research indicates that players in all positions exhibit significantly higher average speeds and greater walking distances in the first half than the second half of matches, reflecting superior physical capacity earlier in the match (34). In addition, tactical adjustments may further contribute to a contraction of passing networks and a reduction in spatial exploration.

## 5 Conclusion

This study utilized social network analysis, with a specific focus on pitch-passing, to analyze passing behavior in the UEFA Women's Euro 2025 under four situational variables. By dividing the pitch into finer spatial zones, the analysis placed particular emphasis on the role of spatial scale in shaping passing interactions. This methodological approach revealed pronounced spatial heterogeneity in pitch-passing metrics under different match situations in elite women's football.

The results demonstrate that winning teams are better able to establish more efficient mediating links, with a greater emphasis on flank-area utilization. This variation in performance is not primarily driven by the quantity or density of passing interactions, but rather determined by the structural organization and functional roles of specific pitch regions within the passing network. From the group to the knockout, the team's passing hub shifted from the defensive third to the middle third and its transitional zones. Additionally, improved local stability and circulation were established in the attacking third. Competitive pressure primarily reshapes the functional contribution of distinct spatial regions to ball progression and network accessibility. Leading teams often establish structured possession corridors within the defensive third to control risk, whereas trailing teams shift activity toward central areas, showing higher midfield intensity but failing to achieve stable local control. Overall, score state shapes both the intensity of play and the spatial organization of team connectivity. A significant decline in the physical capability of the pitch-passing networks was observed. In the 1st half, passing distribution was more extensive, with higher structural integration and coherence of passing clusters. In contrast, overall connectivity and local intensity of the passing network were significantly reduced in the 2nd half.

Coaches are encouraged to emphasize the use of the flanks through drills incorporating diagonal runs, wide-area link-up play, and rapid passing along the sideline. Through small-sided games and restricted training exercises in the final third, designed to promote quick decision-making and reduce unnecessary intermediate passes. Coaches may incorporate structured drills based on triangular passing patterns to enhance local connectivity and support sustained offensive pressure. Together, these zone-specific insights enable practitioners to design training tasks that are closely aligned with match-derived spatial patterns, thereby improving the team's ability to create and exploit high-value attacking opportunities in elite women's football.

## 6 Limitations and further research

Some limitations in the present study arise from data-related and methodological constraints that are common in football network analysis research. For instance, the four situational variables (match outcome, competition stage, score state, and match period) were analyzed independently, and interaction effects were not examined. This decision was primarily driven by the limited sample size (31 matches), as cross-classification would produce small and unbalanced subgroups, thereby reducing statistical reliability. In addition, the use of event-based passing data inherently limits the inclusion of off-ball behaviors and defensive interactions, which are also important components of overall team organization.

Beyond these shared constraints, several limitations are specific to the present study. First, only successful passes were included in the network construction, excluding unsuccessful attempts. While this approach is common in network-based analyzes, it may result in a partial loss of information and does not fully capture risk-taking behavior in passing. Second, the score state was categorized into broad groups (leading vs. trailing), which may not reflect the full complexity of match situations. More detailed score-line distinctions (e.g., 1:0, 1:1, 2:0) could provide a more nuanced understanding of how competitive pressure influences tactical behavior. Third, although spatial zoning was refined, it still represents a discretization of continuous pitch space. Finally, tactical formation was not explicitly incorporated, despite its important role in shaping spatial distribution and passing connectivity.

Despite these limitations, the present study extends previous research in several important ways. Specifically, it integrates a fine-grained spatial division of the pitch with multiple situational variables within a unified analytical framework. This approach enables a more detailed examination of spatial heterogeneity and context-dependent variations in passing networks, particularly in elite women's football, which remains underexplored.

Future research is encouraged to build upon this work by employing larger datasets to examine interaction effects among situational variables, incorporating unsuccessful passes and defensive structures, and applying advanced modeling approaches such as hierarchical or multilayer network models. Additionally, multiple zones can be merged after pitch subdivision to reflect real match conditions. For instance, goalkeepers' passes may span the entire goal area, instead of splitting the penalty area into discrete sub-zones. Such developments would further enhance the robustness and ecological validity of football performance analysis.

## Data Availability

The data used in this study are publicly available from the Statsbomb Open Data repository on GitHub and were accessed and used in accordance with the Statsbomb Public Data User Agreement, hosted at https://github.com/statsbomb/open-data.
